# The bridge between cell survival and cell death: reactive oxygen species-mediated cellular stress

**DOI:** 10.17179/excli2023-6221

**Published:** 2023-06-22

**Authors:** Nese Vardar Acar, Riza Köksal Özgül

**Affiliations:** 1 Department of Pediatric Metabolism, Institute of Child Health, Faculty of Medicine, Hacettepe University, Ankara, Turkey

**Keywords:** redox homeostasis, antioxidant defense systems, Nrf2, oxidative and reductive stress, cell death pathways, mitohormesis

## Abstract

As a requirement of aerobic metabolism, regulation of redox homeostasis is indispensable for the continuity of living homeostasis and life. Since the stability of the redox state is necessary for the maintenance of the biological functions of the cells, the balance between the pro-oxidants, especially ROS and the antioxidant capacity is kept in balance in the cells through antioxidant defense systems. The pleiotropic transcription factor, Nrf2, is the master regulator of the antioxidant defense system. Disruption of redox homeostasis leads to oxidative and reductive stress, bringing about multiple pathophysiological conditions. Oxidative stress characterized by high ROS levels causes oxidative damage to biomolecules and cell death, while reductive stress characterized by low ROS levels disrupt physiological cell functions. The fact that ROS, which were initially attributed as harmful products of aerobic metabolism, at the same time function as signal molecules at non-toxic levels and play a role in the adaptive response called mithormesis points out that ROS have a dose-dependent effect on cell fate determination.

See also Figure 1(Fig. 1).

## Introduction

Our modern understanding of physiological regulation is attributed to physiologists Claude Bernard (1813-1878) and Walter Bradford Cannon (1871-1945), who defined regulation in terms of stability of the internal environment and homeostasis, respectively (Brown and Fee, 2002[26]; Gross, 1998[93]). Bernard proposed the concept of "milieu interieur", which means sustaining of the stability of the internal environment: *"The fixity of the milieu supposes a perfection of the organism such that the external variations are at each instant compensated for and equilibrated. All of the vital mechanisms, however varied they may be, have always one goal, to maintain the uniformity of the conditions of life in the internal environment. The stability of the internal environment is the condition for the free and independent life." *(Gross, 1998[93]). In Bernard's milestone quality, but rather abstract, the concept of the constancy of the “milieu interieur” was clearly and concretely extended by Walter Bradford Cannon in the term “homeostasis” (Cannon, 1929[29]).

The concept of homeostasis, emerged with the development of the conception of "milieu intérieur", expressing by many as the definition of life, appears as a basic feature of biological systems today (Chovatiya and Medzhitov, 2014[40]; Turner, 2017[273]). In the light of current knowledge, it is now known that homeostatic control mechanisms work in the body at all levels including molecular, cellular, tissue, organ and organism (Ayres, 2020[7]; Chovatiya and Medzhitov, 2014[40]).

The cells in multicellular organisms have developed to sense external and internal signals for sustaining cellular homeostasis (Gomes and Blenis, 2015[90]). Stress is a difficult concept to define because it is perplexing and contentious. Because there is not still any precisely defined sensor in the structure of the stress system, it is tough determining which stressors will and will not cause stress (Lu et al., 2021[165]). The using of homeostasis term as a candidate sensor of the stress system is currently very useful and an accepted approach (Lu et al., 2021[165]; Zhou et al., 2019[314]).

**Figure 2:**
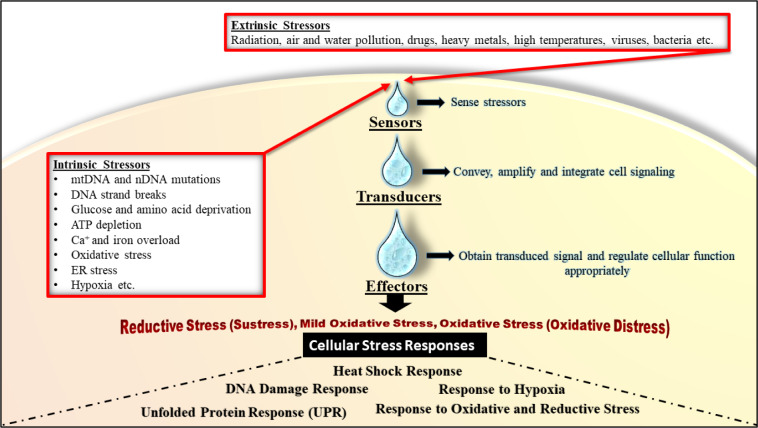
Extrinsic and intrinsic stressors, signal transduction and cellular stress response. The cells are exposed to numerous stress factors, both extrinsic and intrinsic. Stress factors are sensed by the cells, transported, replicated and integrated into the cells. The maintenance of cellular homeostasis is ensured by the regulation of cellular functions through the most appropriate cellular stress response to the transformed stress signal within the cells.

The environmental or intracellular alterations, having the potential to directly or indirectly break down homeostasis, are detected by a cell as stress (Zhou et al., 2019[314]). Stress can be basically divided into two categories: intrinsic and extrinsic (Chovatiya and Medzhitov, 2014[41]; Eisner et al., 2018[69]; Luo and Kraus, 2012[166]) (Figure 2(Fig. 2)). Cells respond to disruptions in their intracellular or extracellular microenvironment due to these stress factors with various mechanisms called "cellular stress responses", that aim to restore cellular homeostasis (Galluzzi et al., 2018[77]; Hotamisligil and Davis, 2016[110]; Twayana and Ravanan, 2018[274]). There are basically four cornerstones of cellular stress responses, involving the coordination of various signaling pathways: (i) macromolecular repair and stabilization, (ii) activation of cell cycle checkpoints, (iii) repartitioning of metabolic energy, and (iv) decisions of programmed cell death. However, it should be noted that programmed cell death (iv) is not a universal characteristic of cellular stress responses like the others (Kültz, 2005[146], 2020[145]). It helps to remove damaged cells and maintain cell integrity by activating when they are exposed to a stress level exceeding their capacity to restore cellular integrity and homeostasis (Gutierrez-Prat et al., 2021[97]; Hotamisligil and Davis, 2016[110]; Kültz, 2020[145]; Twayana and Ravanan, 2018[274]). 

Redox homeostasis, vital for all life functions, is essential for the maintenance of normal cellular homeostasis (Sies et al., 2017[243]). As a requirement of aerobic metabolism, the production of pro-oxidants including free radicals and other reactive species occurs as a result of redox reactions, the most abundant chemical interactions in living cells (Ivanova and Lyublinskaya, 2021[171]; Tauler Riera, 2012[265]). Reactive oxygen species (ROS) is a term used to refer to the most important pro-oxidants produced during metabolic reactions (Phaniendra et al., 2015[214]; Sies and Jones, 2020[244]). Since the stability of the redox state in the cells is necessary for the continuity of the biological functions of the cells, the number of pro-oxidants in the cells is kept in balance with the antioxidant systems (Panieri and Santoro, 2016[206]; Tauler Riera, 2012[265]; Tretter et al., 2021[270]). The pleiotropic transcription factor, the nuclear factor erythroid 2-related factor 2 (Nrf2), plays a fundamental role in the antioxidant stress response (Heurtaux et al., 2022[106]). This dynamic process, which plays a very important role in the formation and maintenance of physiological responses by maintaining the balance between pro-oxidants and antioxidants, is called redox homeostasis (Gambhir et al., 2019[78]; Le Gal et al., 2021[150]). Changes in the balance between pro-oxidants and antioxidants, characterized by an increase or decrease in the redox state, lead to oxidative stress and reductive stress, respectively, resulting in multiple pathophysiological conditions (Xiao and Loscalzo, 2020[290]).

High doses of ROS damage important classes of biological molecules such as nucleic acids, proteins, lipids, and sugars, leading to cell injuries, tissue damage and subsequent pathological conditions (Martemucci et al., 2022[178]). Against high doses of ROS, living organisms develop a series of response mechanisms by using ROS itself as a signal molecule to get rid of the destructive effect of ROS and to ensure cellular integrity (He et al., 2017[105]; Villalpando-Rodriguez and Gibson, 2021[280]). Increasing evidence suggests that high doses of ROS also play a critical role as signaling molecules throughout the entire cell death pathway (He et al., 2017[105]). High doses of ROS have been shown to play a role in inducing various cell death pathways such as autophagy, apoptosis and necrosis. Therefore, high doses of ROS are considered an important factor in promoting cell death (Ghosh et al., 2017[85]; He et al., 2017[105]; Villalpando-Rodriguez and Gibson, 2021[280]). While high levels of ROS are known to be harmful, recent research has shown that mitochondrial stress produced through relatively low and moderate levels of ROS can induce different stress response pathways that ultimately improve the organism's ability to cope with stress through a process called mitohormesis (Hartwick Bjorkman and Oliveira Pereira, 2021[102]; Juan et al., 2021[123]; Palmeira et al., 2019[205]; Ristow and Schmeisser, 2014[228]). As a result, it is apparent that ROS has a dose-dependent effect on cell homeostasis (Zhou et al., 2019[314]).

In this review, we adress how signal transduction occurs in cellular stress responses. Concentrating on ROS-mediated cellular stress, we examine in detail the reactive oxygen species, how redox homeostasis occurs and the transcription factor Nrf2, main regulator of the ROS-mediated cellular stress response. We also discuss the functions/mechanisms of action of ROS at the physiological level and the oxidative and reductive stress associated with pathophysiological conditions. Finally, we emphasize the importance of mitohormesis and cell death pathways, that occur depending on the dose of ROS in cell homeostasis.

## Signal Transduction in Cellular Stress

Eukaryotic cells have advanced sensing mechanisms and signal transduction systems to restore cellular integrity and homeostasis in response to stress (De Nadal et al., 2011[57]; Lu et al., 2021[165]). Different cellular stresses activate the intracellular signaling pathways that control virtually every regard of cell physiology, leading to the stimulation of stress responses including gene expression, metabolism regulation, cell cycle progression, protein homeostasis, cytoskeletal organization, vesicular trafficking, and modification of enzymatic activities (De Nadal et al., 2011[57]). Cellular stress responses consist of both common responses as in the integrated stress response to multiple stressors and particular cellular stress responses specific to certain stresses (De Nadal et al., 2011[57]; Lu et al., 2021[165]; Nwosu et al., 2022[199]; Wang et al., 2018[281]).

Stress-mediated responses are not actually very different from typical known signal transduction systems (Zhou et al., 2019[314]). Signal transmission in the cellular stress response basically consists of three main components: Sensors, transducers, and effectors (Fasano et al., 2019[71]; Lu et al., 2021[165]; Yano and Morotomi-Yano, 2017[296]; Zhou et al., 2019[314]) (Figure 2(Fig. 2)). Cells are exposed to a wide variety of stress factors and activation of more than one pathway takes place in a cell under stress (De Nadal et al., 2011[57]; Zhou et al., 2019[314]). To survive by coping with these stresses cells have sophisticated and diverse stress sensors including growth factor receptors, cytokine receptors, cyclic adenosine monophosphate (cAMP) receptors, and ion channels (Fasano et al., 2019[71]). However, it should be noted that there is limited information about the mechanisms by which different stresses are originally transmitted to the cell (Zhou et al., 2019[314]). To respond quickly and accurately to both intrinsic and extrinsic stress signals perceived by cells, these signals must be transported to effectors through transducers. Transducers either membrane-bound or soluble, contain molecules that directly regulate downstream effectors (Fasano et al., 2019[71]). Stress is sensed or converted into intracellular responses through various systems such as the electron acceptor/donor nicotinamide adenine dinucleotide (NAD), the anti-oxidant glutathione (GSH), and the energy currency of the cell adenosine triphosphate (ATP). Moreover, most stresses give rise to the activation of second messenger systems that stimulate several intracellular stress-inducible second messengers, including ROS, calcium (Ca^2+^), iron (Fe^++^), and cAMP, that activate different effector systems (Zhou et al., 2019[314]). The main effectors of signal transduction pathways are transcription factors, along with other factors that assist the coordinated gene expression. However, in situations where fast-acting responses are required, fast responses such as downregulation of translation, the use of previously transcribed or translated proteins, and the physical regulation of ion channels and transporters can also be created (Brown, 2020[25]; De Nadal et al., 2011[57]; Fasano et al., 2019[71]; Yano and Morotomi-Yano, 2017[296]; Zhou et al., 2019[314]) (Figure 2(Fig. 2)).

Glucose deprivation in the cell is one of the examples of signal transduction in the cellular stress response. In case of glucose deficiency, ATP production decreases, AMP/ATP ratio increases and ROS are overproduced (Zhao et al., 2017[312]). This increase in AMP/ATP ratio is sensed by adenosine monophosphate (AMP)-activated kinase (AMPK) and, as a result of activation of AMPK kinase, this glucose deprivation or energy crisis signal is transmitted via phosphorylation processes to multiple downstream protein targets that act as effectors of AMPK signaling (Kahn et al., 2005[125]). For example, lipid and cholesterol synthesis can be suppressed by the phosphorylation of acetyl-CoA carboxylase 1 (ACC1) and sterol regulatory element binding protein 1c (SREBP1c) by AMPK (Hardie and Pan, 2002[100]; Li et al., 2011[161]). Autophagy of damaged mitochondria and mitochondria biogenesis can be enhanced by phosphorylation of unc-51 like autophagy activating kinase 1 (ULK1) (Egan et al., 2011[67]). Phosphorylation of Rab guanosine triphosphatase-activating protein (RabGAP) TBC1D1 by AMPK can increase glucose uptake by promoting cell membrane translocation of GLUT4 (Pehmøller et al., 2009[208]). Consequently, AMPK activation increases catabolism to provide more energy, while slowing anabolism to prevent overconsumption of ATP. Thus, cells can maintain cellular homeostasis due to this cellular stress response they create against glucose restriction and energy deficiency (Kahn et al., 2005[125]).

Cellular stress response against DNA damage caused by ultraviolet (UV) is another example of stress signal transmission in the cells. UV light is one of the most studied DNA-damaging agents (Cockell and Horneck, 2001[43]). Exposure to UV radiation can cause both directly DNA damage and indirectly cause DNA damage as UV light can cause ROS production in the cells (Batista et al., 2009[13]; Kuluncsics et al., 1999[147]). Certain genome changes (e.g.; mismatched base pairs, single-stranded DNA breaks, double-stranded DNA breaks) in DNA damage response are recognized by DNA sensor molecules (e.g.; replication protein A (RPA), meiotic recombination 11 (MRE11)/DNA repair protein Rad50 (RAD50)/Nijmegen breakage syndrome 1 (NBS1) (MRN) complex. This signal is received by transducers with proteins (e.g.; ataxia telangiectasia mutated (ATM), ATM- and Rad3-Related (ATR), DNA-dependent protein kinase (DNA-PKcs), phosphorylated form of histone H2AX protein encoded by *H2AFx* gene (γH2AX)) that accumulate in the detected damaged areas and transmit the signal. Transducers transfer this signal to effector molecules (e.g.; checkpoint kinase 2 (CHK2), checkpoint kinase 1 (CHK1), p53, DNA repair protein RAD51 (RAD51), breast cancer type 1 susceptibility protein (BRCA1)) (Ciccia and Elledge, 2010[42]; Giglia-Mari et al., 2011[88]; Petrini and Stracker, 2003[212]; Zhou and Elledge, 2000[313]). Thus, genomic stability is maintained through a mechanism consisting of a number of multiple signal transduction pathways such as DNA repair, transcription regulation, cell cycle control, or apoptosis (Giglia-Mari et al., 2011[88]; Jackson and Bartek, 2009[117]; Zhou and Elledge, 2000[313]).

Two points are noteworthy in stress signal transmission: i) ROS increase is a direct or indirect effect that occurs in response to all stresses as given above examples in the stress signal transduction (Zhou et al., 2019[314]). ii) The existence of a dose-dependent stress response, as understood by the evolution of the stress system framework (Lu et al., 2021[165]; Lushchak and Storey, 2021[170]; Zhou et al., 2019[314]). As a result, it is clearly seen that the ROS signal is a signal that should not be ignored in the life and death decision of the cell in almost all stress responses. It is also transpired that cellular stress responses are generated against a variety of stresses through the activation of different types of target proteins capable of inducing cell survival or cell death in a dose-dependent manner (Amici et al., 2022[3]; Brown, 2020[25]; Kourtis and Tavernarakis, 2011[143]; Lu et al., 2021[165]; Simmons et al., 2009[245]; Twayana and Ravanan, 2018[274]; Zhou et al., 2019[314]) (Figure 2(Fig. 2)).

## ROS-Mediated Cellular Stress and Cell Response

For the nature of aerobic metabolism, mammalian cells use a series of oxidation and reduction (redox) reactions to produce energy and synthesize necessary cellular components from nutrients to maintain their biological functions (Ivanova and Lyublinskaya, 2021[116]; Xiao and Loscalzo, 2020[290]). Oxidants, consisting of free radicals and other reactive species, such as ROS which include various molecular oxygen derivatives, are constantly produced in the cell as a by-product of normal aerobic metabolism (Lennicke and Cochemé, 2021[156]; Tauler Riera, 2012[265]; Xiao and Loscalzo, 2020[290]). Cells have developed antioxidant defense systems to prevent oxidative distress due to harmful ROS accumulation (Tretter et al., 2021[270]). In this way, the redox state between pro-oxidants and antioxidants is preserved in the cell and many cellular biological processes necessary for the continuity of the biological functions of the cells are carried out. However, as a result of the deterioration of redox homeostasis in the cell, redox stresses called oxidative and reductive stresses associated with many pathological conditions occur (Xiao and Loscalzo, 2020[290]). Unlike oxidative stress, oxidative stress response, and oxidative stress damage, characterized by excessive ROS production, reductive stress is not a well-known phenomenon and research on this subject continues rapidly (Singh et al., 2015[247]; Xiao and Loscalzo, 2020[290]).

In the light of current information, it is reported that the cell is exposed to reductive stress (sustress or inadequate stress), eustress (oxidative eustress), mild oxidative stress, oxidative stress (oxidative distress) and strong oxidative stress (necrotic stress) (Lu et al., 2021[165]; Lushchak and Storey, 2021[170]; Zhou et al., 2019[314]). Although there is information about the classification of oxidative stress according to its intensity, further definitive studies are needed (Lushchak and Storey, 2021[170]; Zhou et al., 2019[314]). Another proposed classification of oxidative and reductive stress is the time-based classification. A short-term increase in ROS levels with certain functional consequences is called “acute oxidative stress”, and a long-term increase is called “chronic oxidative stress” (Lushchak and Storey, 2021[169]). Both acute and chronic oxidative stress can affect living organisms differently, causing more or less significant damage to cells and can lead to cell death via apoptosis or necrosis if cellular homeostatic control is not regained. It has been suggested that the “acute” and “chronic” approaches proposed for oxidative stress can also be applied to reductive stress. However, it is reported that the concept of reductive stress is not fully developed methodologically and has not been explained comprehensively (Lushchak and Storey, 2021[170]).

In this article, oxidative and reductive stress classifications that cause pathological conditions will be evaluated as a whole and discussed. In this section, after discussing pro-oxidant, antioxidants and regulation of redox homeostasis via Nrf2, the regulations and disorders in cell homeostasis in physiological and pathological redox states will be emphasized.

### ROS

The relationship of higher eukaryotic aerobic organisms with oxygen is highlighted by the term "oxygen paradox". They cannot exist without oxygen, but oxygen is dangerous for their existence because of its nature which could transform into oxygen radicals and other reactive oxygen species, that could give rise to damage to cell, organs, and organisms (Ursini and Davies, 1995[275]). Under normal conditions, more than 90 % of the O_2_ consumed in living organisms is used in ETC by reducing it to H_2_O. The remaining small amount of consumed O_2_ is produced by monovalent reduction of O_2_, producing reactive intermediates containing both free radical and non-radical species (Kohen and Nyska, 2002[137]; Lushchak, 2014[168]; Lushchak and Semchyshyn, 2012[169]).

Free radicals, products of normal cellular metabolism, are atoms or molecules that contain one or more unpaired electrons in an outer atomic orbital or molecular orbital and are capable of independent existence (Halliwell and Gutteridge, 2015[98]; Phaniendra et al., 2015[214]). Free radicals are very unstable, short-lived and highly reactive due to their unpaired electron(s) that this feature is liable for chain reactions (Phaniendra et al., 2015[214]). To maintain molecular stability and form a stable compound, free radicals act as oxidizing or reducing agents, trying to bond with other molecules, atoms or even individual electrons, and donate or accept an electron from others (Halliwell and Gutteridge, 2015[98]). Free radicals are generally formed by homolytic cleavage of the covalent bond, single-electron oxidation or reduction of an atom or molecule (Halliwell and Gutteridge, 2015[98]). Free radical-specific chain reactions are generally divided into three categories: 1) Initiation; reactions that result in a net increase in the number of free radicals. These reactions can contain homolytic cleavage of a covalent bond, oxidation or reduction. 2) Propagation; propagation reactions in which the number of radicals does not change. 3) Termination; termination or disproportionation reactions that result in a net reduction in the number of free radicals. Real termination reactions arise from the interaction of two radicals, but non-radical antioxidants can delay the propagation of radical reactions by causing the production of radical species with much lower reactivity (Kehrer and Klotz, 2015[130]; Khan et al., 2018[125]; Kunath and Moosmann, 2020[148]).

Non-radical reactive intermediates are more stable than free radicals, but non-radical reactive intermediates can easily cause free radical reactions in living organisms (Genestra, 2007[82]). Radical interactions with non-radicals often involve the radical donating its electron or taking an electron from the non-radical molecule or simply joining a non-radical molecule (Halliwell and Gutteridge, 2015[98]).

The widespread availability of oxygen in biological systems causes oxygen-centered radicals to be the most common species. However, in the course of routine cellular activities of living cells, not only oxygen-centered radicals are generated, but also various reactive chemical species, including nitrogen, sulfur, carbonyl, halogen or selenium centered (Halliwell and Gutteridge, 2015[98]; Kehrer and Klotz, 2015[130]; Lushchak, 2014[168]; Martemucci et al., 2022[178]; Phaniendra et al., 2015[214]; Tanaka and Vécsei, 2020[261]) (Table 1(Tab. 1); References in Table 1: Phaniendra et al., 2015[214]; Martemucci et al., 2022[178]; Tanaka and Vécsei, 2020[261]).

The most important class of radicals produced in living systems is ROS with a unique electronic configuration, are produced from diatomic oxygen and are defined as oxygen-containing reactive species. All oxygen radicals are ROS, but not all ROS are oxygen radicals because ROS are used as a term to refer to both free radicals and other non-radical reactive species (Bhat et al., 2015[17]; Cooper, 2018[46]; Li et al., 2016[158]).

ROS production is mainly of exogenous and endogenous origin via enzymatic and non-enzymatic reactions (Lee et al., 2019[151]; Pham-Huy et al., 2008[213]; Pizzino et al., 2017[217]; Sharifi-Rad et al., 2020[235]; Xie et al., 2016[292]). Endogenous activities are the main source of ROS in living organisms (Xie et al., 2016[292]). Mitochondria are the primary endogenous source of ROS in mammalian cells, as ROS are a by-product of oxidative phosphorylation, the reaction in which energy homeostasis is achieved in living cells (Boveris and Cadenas, 2000[23]). In particular, ETC complexes I (NADH-ubiquinone oxidoreductase) and III (ubiquinol-cytochrome c oxidoreductase) are key mitochondrial regions involved in ROS production (Quinlan et al., 2013[218]). ROS production is also carried out in Complex II (Orr et al., 2012[203]; Quinlan et al., 2013[218]). The nicotinamide adenine dinucleotide phosphate oxidase (NOX) family of membrane-bound enzymes is another major endogenous source of ROS (Meitzler et al., 2014[183]). ROS production is also seen in other cellular components such as plasma and nuclear membranes, endoplasmic reticulum (ER), lysosome, peroxisome and cytoplasm (Di Meo et al., 2016[58]). Exogenous sources such as smoking, alcohol, pollutants, drugs or toxins, heavy metal ions, xenobiotics, chemotherapy, UV radiation, nutritional deficiency, exercise, can promote to increase in ROS generation in cells (Lee et al., 2019[151]; Pham-Huy et al., 2008[213]; Pizzino et al., 2017[217]; Sharifi-Rad et al., 2020[235]; Xie et al., 2016[292]).

### Antioxidant defense system in the regulation of redox homeostasis

The equilibrium between the rate and amount of production of pro-oxidants and their elimination over time forms the basis of redox homeostasis. Redox homeostasis is an indispensable requirement for aerobic organisms (Panieri and Santoro, 2016[206]). For this reason, cells use a range of non-enzymatic and enzymatic antioxidant defense systems that work synergistically and in combination with each other to maintain optimal ROS levels (Tripathy and Mohanty, 2017[271]; Zoccarato et al., 2022[319]). Antioxidants are regulated at the level of both mRNA expression and protein enzymatic activity, providing effective quantitative, temporal and spatial management of intracellular ROS (Kong and Chandel, 2018[139]).

**Figure 3:**
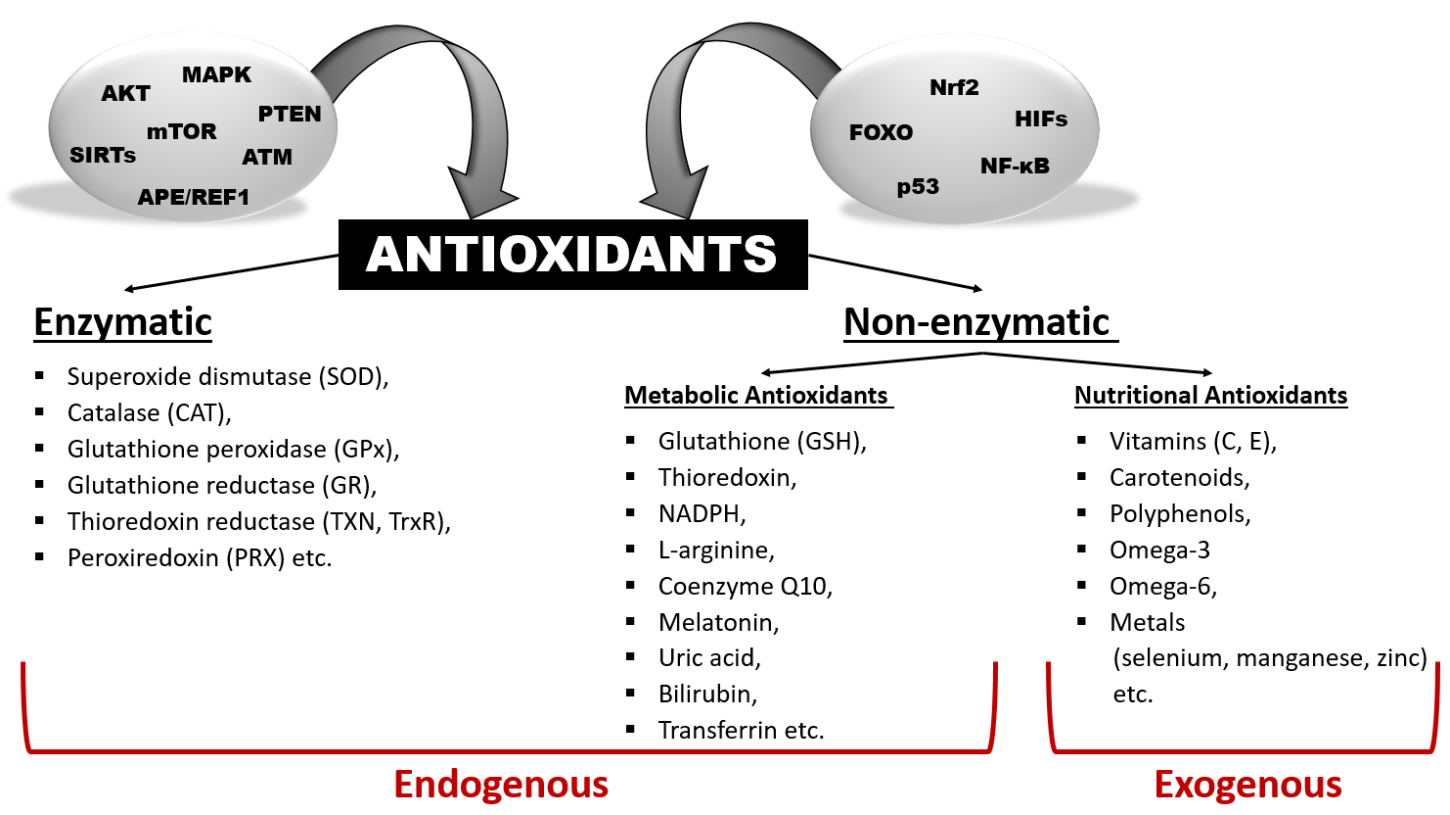
Antioxidant defense systems. Redox balance is tightly controlled through many enzymes and transcription factors, which are directly or indirectly mediate redox homeostasis, and enzymatic and non-enzymatic antioxidants. (Abbreviations: MAPK, mitogen-activating protein kinase; AKT, protein kinase B; APE/REF1, apurinic/apyrimidinic endonuclease 1/redox factor 1; ATM, ataxia-telangiectasia mutated kinase; mTOR, mammalian target of rapamycin; PTEN, phosphate and tensin homologue; SIRTs, sirtuins; FOXO, forkhead box O; NF-κB, nuclear factor-kappa B; p53, tumor suppressor p53; HIFs, hypoxia inducible factors; Nrf2, nuclear factor erythroid 2-related factor 2).

Antioxidants arise from either endogenous or exogenous sources (George and Abrahamse, 2020[83]; Pham-Huy et al., 2008[213]). Endogenous antioxidants produced by metabolism can be classified into two main groups: enzymatic (e.g. superoxide dismutase (SOD), catalase (CAT), thioredoxin, etc.) and non-enzymatic antioxidants. Non-enzymatic antioxidants can be divided into two subgroups: metabolic antioxidants and nutritional antioxidants. Metabolic antioxidants (e.g. bilirubin, coenzyme Q10, melatonin, uric acid etc.) appertain to endogenous antioxidants because they are generated by metabolism in the body. Nutritional antioxidants (e.g. carotenoids, flavonoids, polyphenols, and vitamins C and E, etc.) appertain to exogenous antioxidants because they are compounds that cannot be generated in the body and must be provided via nutrition (George and Abrahamse, 2020[83]; Martemucci et al., 2022[178]; Pham-Huy et al., 2008[213]) (Figure 3(Fig. 3)).

The antioxidant defense system operates in an organized manner to maintain the cellular level of ROS: a) Blocking the initial production of free radicals, b) Removal of pro-oxidants, c) Conversion of pro-oxidants to less toxic compounds, d) Blocking the secondary production of toxic metabolites or inflammatory mediators, e) Termination of chain reactions of secondary pro-oxidants, f) Ensuring the repair of molecular damage caused by pro-oxidants (Tripathy and Mohanty, 2017[271]). In general, antioxidant molecules in living systems act with defense strategies at different levels, namely prevention, interception, and repair (Bhattacharya, 2015[18]; Mirończuk-Chodakowska et al., 2018[188]; Sies, 1993[242]; Sies et al., 2017[243]). On the basis of the line of defense, antioxidants can be categorized in terms of their functions (Ighodaro and Akinloye, 2018[112]; Mirończuk-Chodakowska et al., 2018[188]; Noguchi et al., 2000[198]). First line of defense antioxidants (e.g. SOD, CAT, GPx, transferrin ve seruloplazmin, etc.) act to suppress or prevent the formation of free radicals or reactive species in cells through rapidly neutralizing any molecule that has the potential to turn into a free radical or any free radical that has the ability to trigger the production of other radicals (Ighodaro and Akinloye, 2018[112]; Niki, 2010[194]). Second line of defense antioxidants (e.g. vitamin C, uric acid, albumin, and vitamin E, etc.) scavenge free radicals through suppressing chain initiation and/or stopping chain propagation reactions. In this process, they neutralize or scavenge free radicals by serving electrons to them and become free radicals themselves, possessing less harmful effects. As a result, they are easily neutralized by other antioxidants in this group, rendering them completely harmless (Ighodaro and Akinloye, 2018[112]; Noguchi et al., 2000[198]). The third line of defense antioxidants (e.g. DNA repair enzyme systems-polymerases, glycosylases and nucleases; proteolytic enzymes-proteinases, proteases and peptidases), that come into play after free radical damage has occurred, repair the damage caused by free radicals in biomolecules and remove oxidized or damaged DNA, proteins and lipids to prevent their toxic accumulation in the cell (Ighodaro and Akinloye, 2018[112]; Noguchi et al., 2000[198]). Moreover, the adaptation mechanism function, where appropriate antioxidants are generated at the right time and transferred to the accurate site at the adequate concentration, can serve as a fourth line defense (Ighodaro and Akinloye, 2018[112]; Niki, 2010[194]; Noguchi et al., 2000[198]). There is also evidence to suggest that some antioxidants act as a cellular signaling precursor (Niki, 2010[194]). For example, GSH may play crucial functions in cell signaling through at least two mechanisms, protein S-glutathionylation and cysteine S-nitrosylation (Zhang and Forman, 2012[306]).

In addition to antioxidants, the complex control of cellular ROS homeostasis is directly or indirectly mediated by many enzymes (e.g. mitogen-activating protein kinase (MAPK), ataxia-telangiectasia mutated (ATM) protein kinase, phosphate and tensin homolog (PTEN) and sirtuins (SIRTs), etc.) and transcription factors (e.g. members of the forkhead box O (FOXO) family, hypoxia inducible factors (HIFs) and nuclear factor erythroid 2-related factor 2 (Nrf2), etc.) (Bigarella et al., 2014[20]; Bonello et al., 2007[22]; Corcoran and Cotter, 2013[47]; Diao et al., 2010[59]; Klotz et al., 2015[134]; Kwon et al., 2004[149]; Lee et al., 2002[154]; Singh et al., 2018[246]; Yamamoto et al., 2018[293]; Zhang et al., 2018[310]) (Figure 3(Fig. 3)). Acting as redox sensors, these molecules detect changes in ROS levels and enable the initiation of an appropriate cellular response that induces a variety of cellular processes such as antioxidant responses, gene transcription, differentiation, cell growth, cell proliferation and apoptosis (Bigarella et al., 2014[20]; Lee et al., 2019[151]). With these complex regulations, redox balance is provided and the continuity of cell homeostasis is ensured (Barrera et al., 2021[11]; Bigarella et al., 2014[20]; Boas et al., 2021[21]; Lee et al., 2019[151]).

Although it is known that the regulation of cellular redox homeostasis is provided by the coordinated and controlled regulations of many molecular pathways and molecules in the cell, numerous data show that Nrf2, defined as the main sensor of oxidative stress, is one of the most powerful intracellular antioxidant stress pathways (Chen et al., 2015[32]; Lee et al., 2019[151]; Wang et al., 2023[283]; Zucker et al., 2014[320]).

### The master regulator of redox homeostasis: Nrf2

**Figure 4:**
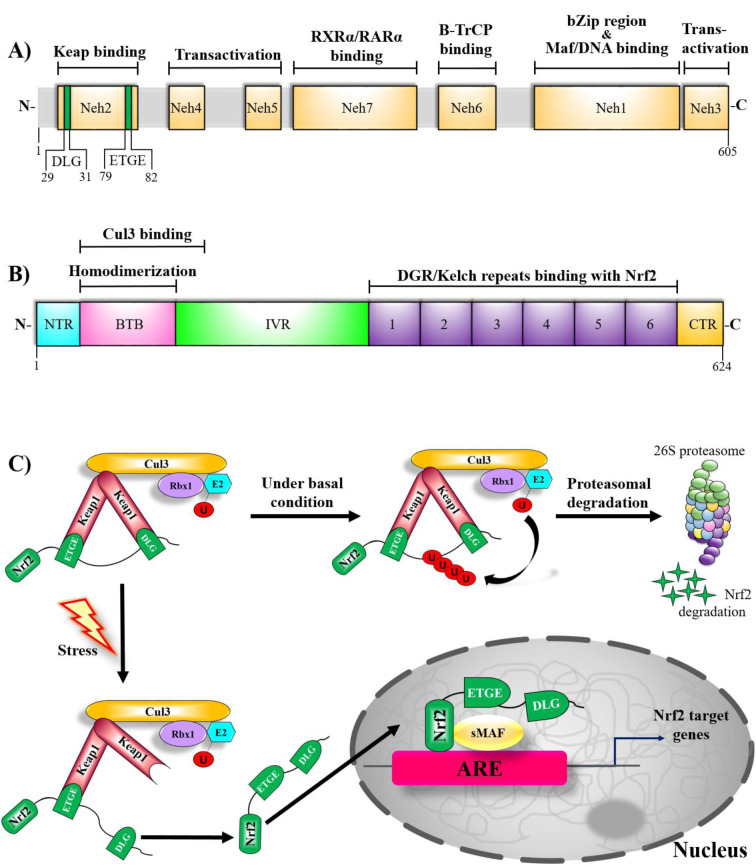
Domain structure of human Nrf2 and Keap1 and schematic representation of the Nrf2/Keap1 signaling mechanism. A) Nrf2, a 605 amino acid protein, comprises seven functional domains called Neh1-Neh7. The N-terminal domain Neh2 contains two motifs, DLG and ETGE, which are responsible for binding KEAP1 homodimer for performing ubiquitin-dependent proteasomal degradation of Nrf2. The Neh4 and Neh5 domains recruit transcriptional co-activators, CREB-binding protein (CBP), and/or repressor-associated coactivator (RAC) for the transactivation activity of NRF2. The Neh7 domain binds retinoid X (RXR) and retinoic acid (RAR) receptors that mediates repression of Nrf2. The Neh6 domain contains two motifs (DSGIS and DSAPGS) interacting with β-transducin repeat-containing protein (β-TrCP) for the β-TrCP-mediated proteasomal degradation. The Neh1, containing serine-rich domain, is responsible for dimerization with small musculoaponeurotic fibrosarcoma (Maf), which is the heterodimeric partner for Nrf2 to recognize the ARE sequence in target gene promoters. The C-terminal domain, Neh3, is a transcriptional co-activator that recruits chromodomain helicase DNA-binding domain protein 6 (CHD6). B) A 624-amino acid Keap1, the repressor of Nrf2, contains three functional domains in addition to the N-terminal and C-terminal domains. The broad complex/tram track/bric-a-brac (BTB) domain regulates Keap1 homodimerization and interaction with the Cul3-based ubiquitin E3 ligase complex for NRF2 ubiquitination. Intervening region (IVR) domain acts as a sensor for NRF2 inducers through highly-reactive cysteine residues. the double-glycine repeats (DGR)/Kelch domain is important for binding with the Neh2 domain of NRF2. C) Under basal conditions, Nrf2 binds to Keap1 via ETGE and DLG motifs in the cytosol and activates Cul3-mediated ubiquitination through interacting with the Cul3-RBX1 E3 ubiquitin ligase. Thus, ubiquitinated Nrf2 is degraded by the 26S proteasome. Under stressful conditions, Nrf2 dissociates from Keap1 and accumulates due to conformational changes in Keap1 as a result of modifications in cysteine residues of Keap1.Then, Nrf2 translocates into the nucleus, forms a heterodimer with sMaf proteins and binds to ARE to initiate the transcription of Nrf2 target genes. (Abbreviations: Nrf2, nuclear factor erythroid 2-related factor 2; Keap1, Kelch-like ECH-associated protein 1; Cul3, Cullin 3; Rbx1, Ring box 1; E2, ubiquitin-conjugating enzyme; ARE, antioxidant response elemen; U, Ubiquitin)

**Figure 5:**
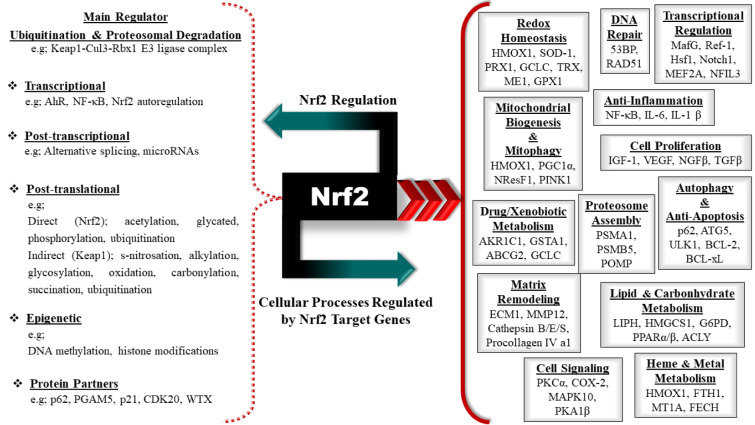
Regulation of Nrf2 and Nrf2 target genes. The target genes and cellular processes regulated by Nrf2 are illustrated with instances. Also, the regulation of this complex and multifunctional transcription factor is also explained with examples. (Abbreviations: Keap1-Cul3-Rbx1, Kelch-like ECH-associated protein 1-Cullin 3-Ring box 1; AhR, aryl hydrocarbon receptor; NF-κB, nuclear factor-kappa B; PGAM5, PGAM family member 5, mitochondrial serine/threonine protein phosphatase; CDK20, cyclin dependent kinase 20; WTX, Wilms tumor gene on the X chromosome; HMOX1, heme oxygenase 1; SOD-1, superoxide dismutase-1; PRX1, peroxiredoxin 1; GCLC, glutamate-cysteine ligase catalytic subunit; TRX, thioredoxin; ME1, malic enzyme 1; GPX1, glutathione peroxidase 1; 53BP, p53-binding protein 1; RAD51, DNA repair protein RAD51 homolog 1; MafG, V-Maf avian musculoaponeurotic fibrosarcoma oncogene homolog G; Ref-1, redox effector factor-1; Hsf1, heat shock transcription factor 1; Notch1, neurogenic locus notch homolog protein 1; MEF2A, myocyte enhancer factor 2; NFIL3, nuclear factor interleukin (IL)-3 regulated; PGC1α, peroxisome proliferator-activated receptor gamma activator 1 alpha; NResF1, nuclear respiratory factor 1; PINK1, PTEN-induced kinase 1; IL-6, Interleukin 6; IL-1 β, Interleukin-1 beta; IGF-1, growth factors [insulin like growth factor-1; VEGFα, vascular endothelial growth factor alpha; NGFβ, nerve growth factor beta; TGFβ, transforming growth factor beta; AKR1C1, aldo-keto reductase family 1 member C1; GSTA1, glutathione S-transferase alpha 1; ABCG2, ATP binding cassette subfamily G member 2 (Junior blood group); GCLC, glutamate-cysteine ligase catalytic subunit; PSMA1, proteasome subunit alpha 1; PSMB5, proteasome subunit beta 5; POMP, proteasome maturation protein; ATG5, autophagy-related 5; ULK1, Unc-51-like kinase 1; BCL-2, B-cell lymphoma 2; Bcl-xL, B-cell lymphoma-extra large; ECM1, extracellular matrix protein 1; MMP12, matrix metallopeptidase 12; LIPH, lipase member H; HMGCS1, 3-hydroxy-3-methylglutaryl-CoA synthase 1; G6PD, glucose-6-phosphate dehydrogenase; PPARα/β, peroxisome proliferator-activated receptor alpha/beta; ACLY, ATP citrate lyase; PKCα, protein kinase C, alpha; COX-2, cytochrome c oxidase subunit II; MAPK10, mitogen-activated protein kinase 10; PKA1β, protein kinase a1 beta; FTH1, ferritin heavy chain 1; MT1A, metallothionein 1A; FECH, ferrochelatase.

In addition to the role of the transcription factor Nrf2 as the master regulator of redox homeostasis, Nrf2 is a pleiotropic transcription factor that regulates the expression of more than 500 different genes involved in numerous cellular processes, including phase I - III drug/xenobiotic metabolism, protein homeostasis, ubiquitin system and autophagy, DNA repair, carbohydrate and lipid metabolism, iron homeostasis, transcriptional regulation and mitochondrial function (reviewed in detail and reported by (Audousset et al., 2021[5]; Chen, 2021[35]; Chen and Maltagliati, 2018[36]; Cuadrado et al., 2018[49]; Dodson et al., 2019[65]; Gutiérrez-Cuevas et al., 2022[96]; Heurtaux et al., 2022[106]; Menegon et al., 2016[184]; Paladino et al., 2018[204]; Zgorzynska et al., 2021[303])) (Figure 5). Nrf2, described as a major sensor of oxidative stress in the cell, belongs to the cap ´n´ collar (CNC) transcription factors family with a basic leucine zipper region (bZip) and interacts with the cysteine thiol groups of the Kelch-like ECH-associated protein 1 (Keap1), an oxidative stress sensor (Dinkova-Kostova et al., 2002[61]; Itoh et al., 1997[113]; Itoh et al., 1999[114]; Moi et al., 1994[189]). Nrf2 possesses conserved seven functional Nrf2-ECH homology (Neh) domains (Neh1-7), important in its regulation (Itoh et al., 1999[114]). Neh1 includes a bZIP structure, crucial for Nrf2 dimerization with small muscle aponeurosis fibromatous (sMAF) proteins and DNA binding. Moreover, it regulates Nrf2 protein stability through interacting with UbcM2, the E2 ubiquitin-conjugating enzyme (Keum and Choi, 2014[131]). Neh1 also comprises a nuclear localization signal (NLS) fundamental for the nuclear translocation of Nrf2 (Theodore et al., 2008[266]). Neh2, containing lysine residues, is responsible for Keap1-mediated proteasomal degradation of Nrf2, binds Nrf2 to Keap1 and contains two distinct motifs, DLG and ETGE (Katoh et al., 2005[128]; McMahon et al., 2006[181]; Zhang et al., 2004[305]). Neh3, Neh4 and Neh5, required for the transactivation of Nrf2, are transactivator domains that interact with intracellular co-activator molecules (Katoh et al., 2001[128]; Kim et al., 2013;[133] Nioi et al., 2005[195]). Neh6, having a serine-rich region contained in Keap1-independent negative regulation of Nrf2, organizes the stability of Nrf2 (Chowdhry et al., 2013[41]; Rada et al., 2011[219], 2012[220]; Suzuki et al., 2000[258]; Wu et al., 2003[288]). The Neh7 interacts with retinoid X (RXRs) and retinoic acid (RARs) receptors, that prevent the binding of the transcription co-activators to the Neh4 and Neh5, thereby mediating the repression of Nrf2 (Wang et al., 2013[282]) (Figure 4A(Fig. 4)).

The Keap1 protein contains 27 cysteine residues, some of which are accessible for redox oxidation or electrophile conjugation and act as a stress sensor (Dinkova-Kostova et al., 2002[61]; Eggler et al., 2005[68]; Holland and Fishbein, 2010[109]; Kansanen et al., 2013[127]; Magesh et al., 2012[173]; Suzuki et al., 2019[259]; Zhang and Hannink, 2003[304]). Keap1, the repressor of Nrf2, belongs to the Kelch-like family of proteins involving the BTB (broad complex/tram track/bric-a-brac) domain and consists of five domains: 1) the N-terminal region (NTR), 2) the BTB region, 3) an intervening region (IVR), 4) a double-glycine repeat (DGR)/Kelch domain, and 5) the C-terminal region (CTR) (Li et al., 2004[160]). BTB domain is fundamental for homodimerization of Keap1, for interactions with the Cullin 3-Ring box 1 (Cullin-3-Rbx-1) E3 ligase complex (Zipper and Mulcahy, 2002[317]). The IVR domain, with its highly reactive cysteine residues, functions as biochemical sensors of cellular stress and has a nuclear export signal (NES) that regulates the cytoplasmic localization of Keap1 (Dinkova-Kostova et al., 2002[61]; Ogura et al., 2010[200]; Velichkova and Hasson, 2005[279]; Yamamoto et al., 2008[294]). The DGR/Kelch domain comprises six Kelch repeats that act as binding sites for the ETGE motif of the Neh2 domain of Nrf2 and also other protein such as p62 which lead to competitive inhibition of Nrf2 (Itoh et al., 1999[114]; Komatsu et al., 2010[138]; Li et al., 2004[160]; Lo et al., 2006[163]; Tong et al., 2006[269]). Moreover, the DGR and CTR domains, collectively called the DC region, are responsible for the interaction of KEAP1 with Nfr2 (Li et al., 2004[160]; Lo et al., 2006[163]) (Figure 4B(Fig. 4)).

Regulation of Nrf2 occurs mainly by controlling Nrf2 protein levels through ubiquitination and proteasomal degradation (Itoh et al., 2003[115]; Zhao et al., 2014[311]). There are four known ubiquitin ligase systems that are responsible for Keap1-dependent and Keap1-independent Nrf2 activation. The Keap1-Cul3-Rbx1 E3 ligase complex, the first discovered, most studied, and involved in Keap1-dependent Nrf2 activation, is considered the canonical mechanism of negative Nrf2 regulation (Cullinan et al., 2004[51]; Kobayashi et al., 2004[135]; Zhang et al., 2004[305]). Under basal conditions, Keap1 binds to ETGE and DLG motifs in the Neh2 domain of Nrf2 via the Kelch-repeat domain, forming a homodimer resulting in cytoplasmic retention (Itoh et al., 1999[114]; Ogura et al., 2010[200]; Tong et al., 2006[269]). Keap1 acts as a substrate adapter protein for the ubiquitin ligase Cul3/Rbx1, which is responsible for the ubiquitylation and degradation of Nrf2 (Cullinan et al., 2004[51]; Furukawa and Xiong, 2005[74]; Kobayashi et al., 2004[135]; Zhang et al., 2004[305]). The binding of Nrf2 to Keap1 in the cytoplasm brings the Cul3/Rbx1 E3 ubiquitin ligase into the complex and targets Nrf2 for poly-ubiquitination and degradation by the 26S proteasome (Baird et al., 2013[8]) (Figure 4C(Fig. 4)). Nrf2 has a short half-life of approximately 10-30 minutes. Thus, the Keap1-mediated turnover of Nrf2 keeps Nrf2 basal levels extremely low and prevents unnecessary expression of Nrf2 target genes (Nguyen et al., 2003[193]; Stewart et al., 2003[253]).

Pro-oxidants and electrophiles cause electrophilic modification of cysteine residues of Keap1 (Baird et al., 2013[8]; Dinkova-Kostova et al., 2002[61], 2005[62][63]; McMahon et al., 2010[180]). As a result of this modification, ubiquitination and proteasomal degradation of Nrf2 are inhibited by the conformational change in Keap1, and Nrf2 is released. Accumulated free Nrf2 translocates to the nucleus, where it heterodimerizes with small musculo-aponeurotic fibrosarcoma (sMAF) proteins. By binding to the antioxidant response element (ARE), the target DNA region, a stress response is created by activating the transcription of the target genes (Hirotsu et al., 2012[108]; Itoh et al., 1997[113], 2003[115]; Kobayashi et al., 2006[136]; McMahon et al., 2006[181]; Motohashi et al., 2004[190]; Tong et al., 2006[269]) (Figure 4C(Fig. 4)).

Nrf2 can also be regulated in non-canonical pathways by KEAP1-independent mechanisms. Three E3 ubiquitin ligase complexes are known to be included in Keap1-independent Nrf2 degradation: 1) βTrCP-S-phase kinase-associated protein-1 (Skp1)-Cul1-Rbx1, 2) 3-hydroxy-3-methylglutaryl reductase degradation 1 (Hrd1) and 3) Cullin4/damaged DNA binding protein-1/WD Repeat Domain 23 (CUL4/DDB1/WDR23). β-TrCP, serving as substrate recognition subunits for SCFβ-TrCP (Skp1-Cullin1-F-Box protein) E3 ubiquitin ligases, causes Nrf2 ubiquitination and degradation. Glycogen synthase kinase 3 beta (GSK-3β), which can phosphorylate β-TrCP, increasing Nrf2 ubiquitination (Chowdhry et al., 2013[41]; Rada et al., 2011[219]). Hrd1 can interact under reticulum stress conditions with Neh4 and 5 domains and trigger Nrf2 degradation (Wu et al., 2014[289]). CUL4/DDB1/WDR23 was recently discovered to be another E3 ligase of Nrf2. WRD23 binds near the Nrf2 DLG motif and regulates its ubiquitination and degradation, however, its role in Nrf2 stability is still poorly understood (Lo et al., 2017[162]). The most studied mechanism of the non-canonical pathway is Nrf2 activation via the p62/SQSTM1 (sequestosome 1) protein. p62/SQSTM1, an important component of autophagy and a target of Nrf2, binds to KEAP1 and competes with the ETGE motif of Nrf2, resulting in expression of Nrf2 target genes with inhibition of Nrf2 degradation (Komatsu et al., 2010[138]).

The regulation of Nrf2, which controls the expression of a variety of genes and plays a role in the regulation of many molecular signaling pathways, is not limited to these mechanisms. Nrf2 expression and activities are also tightly controlled through transcriptional, post-transcriptional, post-translational, epigenetic, and other protein partners other than p62/SQSTM1 (reviewed in detail and reported by (Basak et al., 2017[12]; Cheng et al., 2016[38]; Dodson et al., 2019[65]; Menegon et al., 2016[184]; Pillai et al., 2022[215]; Shaw and Chattopadhyay, 2020[237]; Tonelli et al., 2018[268]; Zgorzynska et al., 2021[303])) (Figure 5(Fig. 5)).

Abundant evidence demonstrates that Nrf2 dysregulation plays an important role in a wide variety of diseases, including diabetes, cardiovascular diseases, cancer, and neurodegenerative diseases, and the role of Nrf2 can be complex in diseases (Cominacini et al., 2015[44]; Dodson et al., 2019[65]; Esteras et al., 2016[70]; Leinonen et al., 2015[155]; Ngo and Duennwald, 2022[192]; Ying et al., 2016[298]). For example, while Nrf2 activation was detected in some studies on Alzheimer's disease (AD) (Raina et al., 1999[221]; SantaCruz et al., 2004[230]; Schipper et al., 1995[233]; Tanji et al., 2013[263]; Wang et al., 2000[284]), Nrf2 suppression was shown in others (Johnson and Johnson, 2015[120]; Ramsey et al., 2007[224]). In addition to the studies with AD, the study with Parkinson's disease (PD) pointed to Nrf2 activation (Johnson and Johnson, 2015[120]), while the study with amyotrophic lateral sclerosis (ALS) revealed low Nrf2 protein levels (Sarlette et al., 2008[231]). The reason for the detection of these inconsistent findings about Nrf2 in neurodegenerative diseases may be specific to the cell type and brain region or may depend on the stage of the disease under investigation (Dodson et al., 2019[65]; Johnson and Johnson, 2015[120]). Although these contradictory findings, it is an undisputed fact that Nrf2 plays a role in the pathophysiology of neurodegenerative diseases.

### Physiological role of ROS-mediated cell signaling

Under physiological conditions, ROS levels fluctuate in a certain range (the optimal level of ROS), which is generally called in the literature the "steady-state ROS level" (Lushchak, 2011[167]), although there are definitions such as "redox tone" (Sies and Jones, 2020[244]), "redox window” (Yun et al., 2009[302]), "'oxidative eustress" (Sies, 2017[240]). Orginally ROS sensed as the undesirable products of destructive oxidative stress, but it is known today that steady-state level of ROS is vital for the regulation of physiological cellular functions via redox signals and the maintenance of cellular homeostasis (Dickinson and Chang, 2011[60]; Sies and Jones, 2020[244]). Steady-state ROS level perform essential role both as redox-signaling molecules in multifarious pathways taken part in the maintenance of cellular homeostasis and coordinating fundamental transcription factors: including AKT (protein kinase B) kinases, MAPKs (mitogen-activated protein kinases, ATM (ataxia-telangiectasia mutated), mTOR (mammalian target of rapamycin), PTEN (phosphate and tensin homolog), SIRTs (sirtuins) and AMPK (adenosine monophosphate (AMP)-activated kinase), Nrf2/Keap1 (nuclear factor erythroid 2 (NF-E2)-related factor 2/Kelch-like ECH-associated protein 1); NFκB (nuclear factor-κB); HIF-1α (hypoxia-inducible factor1-α); FOXO (forkhead box O transcription factor), p53 (p53- tumor suppressor)) (Bell et al., 2011[15]; Byun et al., 2009[28]; Chen et al., 2009[33], 2010[34]; Dansen et al., 2009[53]; Guo et al., 2010[95]; Hayashi et al., 2015[104]; Hinchy et al., 2018[107]; Lee et al., 2002[154], 2016[153]; Lotem et al., 1996[164]; Nemoto and Finkel, 2002[191]; Takada et al., 2003[260]; Ushio-Fukai et al., 1999[276]; Wang et al., 2014[285]; Zhu et al., 2005[315]; Zmijewski et al., 2010[318]).

The most relevant ROS in maintaining steady-state ROS level under physiological conditions are superoxide anion radical (O_2_^•−^) and H_2_O_2_, respectively (Sies and Jones, 2020[244]). However, it should be noted that H_2_O_2_ is the main redox metabolite that functions in redox sensing, signaling and redox regulation (Marinho et al., 2014[177]). There are detailed data on H_2_O_2_ being the main redox metabolite (Forman et al., 2010[73]; Marinho et al., 2014[177]), to summarize: 1) Up to 1-4 % O_2 _is reduced to O_2_^•−^, the first ROS to form. However, O_2_^•− ^is unstable in aqueous solutions due to its short half-life. Steady-state levels of O_2_^•−^ are achieved by rapidly occurring spontaneous and/or enzyme-mediated dismutation into H_2_O_2_ catalyzed by superoxide dismutases (SOD1-3). Compared to O_2_^•−^, H_2_O_2_ is more stable, 100 times higher than O_2_^•− ^concentration in mitochondria and exhibiting low overall reactivity. 2) H_2_O_2_ shows high selectivity for the thiol group of cysteine residues. Thus, -SH groups of proteins involved in signaling such as phosphatases, kinases and transcription factors containing cysteine residues are specifically oxidized by H_2_O_2_, resulting redox regulation through a series of molecular processes. 3) H_2_O_2_ can move across membranes by passive diffusion or facilitated transport (reviewed in detail and reported by (Forman et al., 2010[73]; Lennicke and Cochemé, 2021[156]; Marinho et al., 2014[177]; Sies, 2017[240]; Sies and Jones, 2020[244]; Sun et al., 2020[256])).

For the maintenance of cell homeostasis by the extraordinarily complex and extensive regulation of these multifarious pathways molecules and essential transcription factors, ROS (especially H_2_O_2_ as described above) participate in numerous and diverse physiological processes such as: proliferation (Lyublinskaya et al., 2015[171]), differentiation (Ji et al., 2010[118]), epigenetic modifications (Bazopoulou et al., 2019[14]) and gene regulation/physiological signaling/metabolism. There are detoxification, electrolyte transport, gluconeogenesis, regulation of epitelial function, neurogenesis, synaptic plasticity, angiogenesis, regulation of heart rhythm and constriction, hematopoiesis, inflammation/innate immunity and lifespan within gene regulation/physiological signaling/metabolism. (Zhang et al., 2019[309]).

### Oxidative and reductive stress: ROS-mediated cellular stress

Redox homeostasis in a cell is achieved through the antioxidant defense system and some redox couples, including glutathione/glutathione disulfide (GSH/GSSG), nicotinamide adenine dinucleotide hydrogen (NADH)/nicotinamide adenine dinucleotide (NAD^+^), nicotinamide adenine dinucleotide phosphate hydrogen (NADPH)/nicotinamide adenine dinucleotide phosphate (NADP^+^), which work in concert with antioxidant enzymes (Chaiswing et al., 2018[31]; Harris and Hansen, 2012[101]; Jones et al., 2004[121]). The NAD and NADP systems, together with the thiol/disulfide systems, play an essential role in the regulation of the redox state. NAD(H) participates in numerous redox reactions as electron carriers. The [NADH]/[NAD^+^] plays a fundamental role in the regulation of redox homeostasis, catabolism, and energy metabolism (Jones and Sies, 2015[122]; Li et al., 2022[159]; Ying, 2006[297]). NADP(H) is structurally similar to NAD(H), but it has different biochemical functions. [NADPH]/[NADP^+^] supplies essential reducing power for anabolism and antioxidant functions (Agledal et al., 2010[1]). NADP(H) regulates cellular redox homeostasis through the enzymatic antioxidant defense systems glutathione system (GSH/GSSG) and thioredoxin system (Trx-SH/Trx-SS) (Jones and Sies, 2015[122]; Li et al., 2022[159]; Xiao et al., 2018[291]).

**Figure 6:**
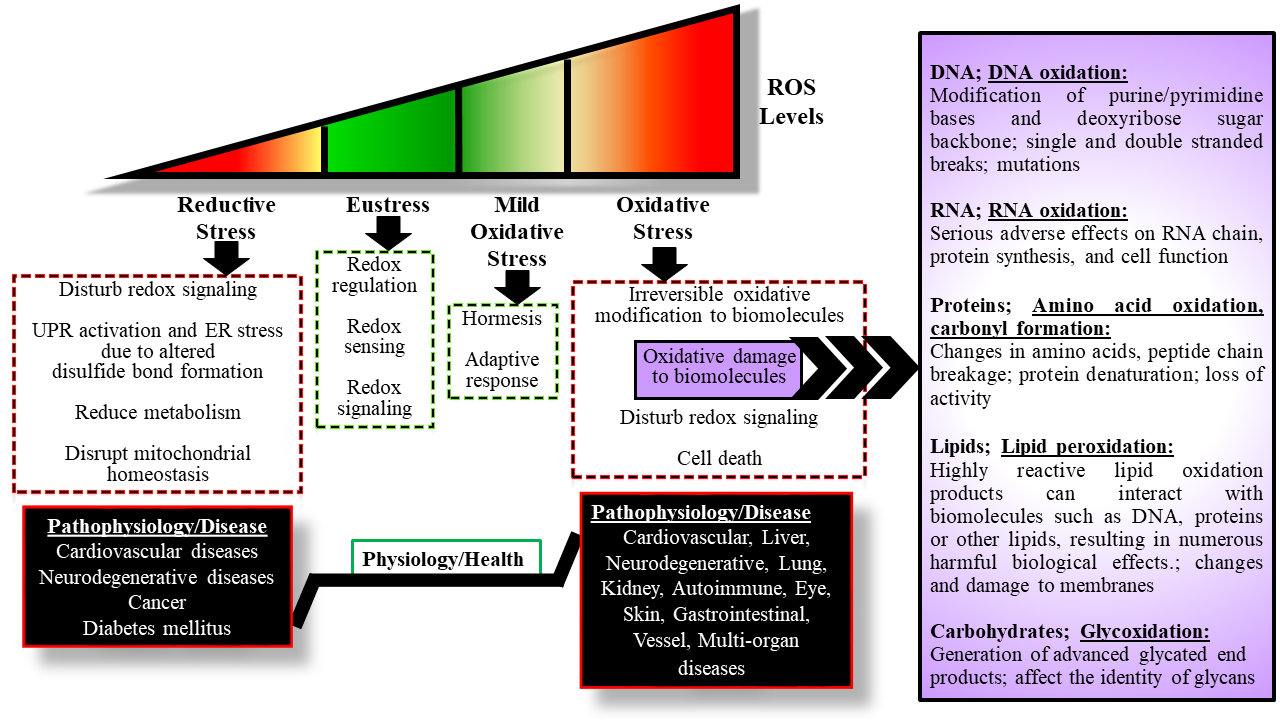
ROS levels are the bridge between cell survival and death response, resulting in physiology/ health and pathology/diseases.

NAD(P)H/NAD^+^ and GSH/GSSG redox couples, the main cellular redox buffers, act as cofactors or substrates in the enzymatic or non-enzymatic neutralization of ROS to provide a comparatively reducing environment in cells. Under normal conditions, these cellular redox buffers have adequate capacity for sustaining physiological levels of cellular oxidants and reductants, referred basal redox buffer capacity (ReBC), where ROS acts as a signaling molecule in the cell (Xiao and Loscalzo, 2020[290]). The ratio of different intracellular electron capture systems, including the antioxidant GSH and electron acceptors/donors NAD(P), is an indicator of the redox state, responsible for cell signal maintenance and cell stress adaptation (Meng et al., 2021[185]; Surai et al., 2021[257]; Zhou et al., 2019[314]) (Figure 6(Fig. 6)). However, changes in the balance between pro-oxidants and antioxidants, characterized by an increase or decrease in the redox state, cause the formation of redox stresses, which are called oxidative stress and reductive stress, respectively (Brewer et al., 2013[24]; Gores et al., 1989[91]; Paniker et al., 1970[207]) (Figure 6(Fig. 6)).

Oxidative stress is described as an imbalance between cellular pro-oxidant levels and antioxidant capacity due to excessive pro-oxidant levels, giving rise to deterioration redox signaling and its control, and/or oxidative damage to cellular components (Pesta and Roden, 2017[211]; Sies, 2019[241]; Xiao and Loscalzo, 2020[290]). Oxidative stress may result from cellular ReBC reduction and/or overproduction of ROS and/or depletion of enzymatic and non-enzymatic antioxidant systems (Pérez-Torres et al., 2017[209]; Xiao and Loscalzo, 2020[290]). Increasing concentrations of ROS can result in oxidative modification of important classes of biological molecules such as nucleic acids, proteins, lipids, and carbohydrates (Riley, 1994[227]) (Figure 6(Fig. 6)).

Oxidatively modified biomolecules can act as genuine signaling molecules. For instance, an electrophilic lipid with low reactivity forms adducts with cysteine residues and alters the cell signaling (Levonen et al., 2004[157]) However, cell and tissue damage with pathological effects may occur if the rate at which oxidatively modified biomolecules are produced by antioxidant and/or repair systems exceeds their removal from biological systems and/or their replacement with fully new functional molecules in biological systems (Davies, 2000[54]). Modifications in biomolecules due to oxidative stress can lead to abnormal cell functions by causing various pathological effects such as damage and deterioration in membrane lipids, structural proteins, enzyme activity, receptor function and transport function, and alteration in gene expression (Butterfield et al., 1998[27]; Davies, 2000[54]). For example, 8-hydroxy-2'-deoxyguanosine (8-OHdG), which is formed as a result of ^•^OH, preferentially oxidizing the guanine base of DNA, is one of the most studied examples of DNA oxidative damage modification (Cooke et al., 2003[45]; Dizdaroglu and Jaruga, 2012[64]). 8-OHdG can affect various mechanisms such as replication and transcription and change the epigenetic profile in the cells (Cooke et al., 2003[45]; Gaillard et al., 2015[75]; O'Hagan et al., 2011[201]). DNA damage has been observed in many diseases, including cardiovascular, inflammatory and neurodegenerative diseases (Cooke et al., 2003[45]; Kosanovic et al., 2021[141]; Kroese and Scheffer, 2014[144]; Mecocci et al., 1998[182]). Another example of oxidative damage is lipid peroxidation, caused by oxidants attacking unsaturated lipids and causing the formation of lipid oxidation products such as 4-hydroxy-2-nonenal (4-HNE), malondialdehyde (MDA), oxylipins, and isoprostanes (Gianazza et al., 2021[86]). 4-HNE, one of the main lipid oxidation products resulting from enzymatic and non-enzymatic oxidative pathways from oxidized phospholipids containing polyunsaturated fatty acid (PUFA) n-6 chains, can increase ROS generation and inflammation, alter cell signaling, and cause cell damage and apoptosis (Ayala et al., 2014[6]; Spickett, 2013[251]). Other example of oxidative damage is protein oxidation, which causes post-translational modifications that alter amino acid and protein composition, structure, charge, hydrophobicity/hydrophilicity, and folding (Dalle‐Donne et al., 2006[52]; Davies, 2005[56], 2016[55]; Gianazza et al., 2007[87]).

In addition, mitochondria are both the primary endogenous source and target of ROS, so oxidative stress is inextricably linked to mitochondrial dysfunction, and there is a vicious circle between oxidative stress and mitochondrial dysfunction. Oxidative stress causes oxidative damage to mitochondrial biomolecules. While this oxidative damage in mitochondria causes mitochondrial dysfunction, there is an increase in ROS production as a result of mitochondrial dysfunction (Hyatt and Powers, 2021[111]; Mancuso et al., 2009[174]; Shokolenko et al., 2014[239]; Soiferman et al., 2014[248]). For example, mitochondrial dysfunction and oxidative stress play a role in the pathomechanism of primary mitochondrial diseases caused by germline mutations in mtDNA and/or nDNA genes that encode OXPHOS structural proteins or mitochondrial proteins of the complex mechanism required to carry out the OXPHOS process (Baker et al., 2022[9]; Hayashi and Cortopassi, 2015[103]; Niyazov et al., 2016[197]; Valenti and Vacca, 2022[277]). However, mitochondrial dysfunction, which occurs as a secondary consequence of the disease pathophysiology, may contribute greatly to the formation of ROS in some disorders such as inborn errors of metabolism (IEM). Several IEMs have been proposed to involve shared pathomechanisms involving mitochondrial dysfunction and increased ROS levels (Mc Guire et al., 2009[179]; Olsen et al., 2015[202]; Richard et al., 2018[226]; Stepien et al., 2017[252]). Considering the importance of redox homeostasis in normal physiology and the devastating effects of impaired redox homeostasis in the cell, it is actually not surprising that oxidative stress is associated with a wide variety of disease pathophysiologies (reviewed in detail and reported by (Kehrer and Klotz, 2015[130]; Lennicke and Cochemé, 2021[156]; Phaniendra et al., 2015[214]; Pisoschi et al., 2021[216]; Pizzino et al., 2017[217]; Rani and Yadav, 2015[225]; Sharifi-Rad et al., 2020[235]; Sharma et al., 2018[236])) (Figure 6(Fig. 6)).

For example, high levels of oxidative damage have been observed in postmortem brain tissues of patients with neurodegenerative diseases, suggesting that oxidative stress plays a role in the formation and/or exacerbation of the distinctive protein inclusions seen in neurodegenerative diseases. In addition, studies are showing that Nrf2 levels, which are activated in response to oxidative stress, may be impaired or insufficient in neurodegenerative diseases (Ngo and Duennwald, 2022[192]). Although studies on the effects of ROS in the pathophysiology of IEMs are at the initial stage compared to ROS-related studies in other diseases, it has been reported that oxidative stress and Nrf2/Keap1 pathway are involved in the pathophysiology of IEMs (Vardar Acar et al., 2021[278]). It is possible to detail examples of oxidative stress and the roles of Nrf2 in disease pathophysiology (Al-Sawaf et al., 2015[2]; Cuadrado et al., 2019[50]; Gambhir et al., 2022[79]; Ngo and Duennwald, 2022[192]; Phaniendra et al., 2015[214]; Pisoschi et al., 2021[216]; Sharifi-Rad et al., 2020[235]; Sharma et al., 2018[236]; Tu et al., 2019[272]).

The concept of reductive stress is not known as comprehensively as oxidative stress, and the mechanisms associated with reductive stress have not been fully elucidated (Rajasekaran, 2020[222]). On the other hand, our understanding of reductive stress has evolved since the concept was first introduced and defined (Gores et al., 1989[91]; Wendel, 1987[286]), by means of an increasing number of studies on reductive stress (Rajasekaran, 2020[222]). In general, reductive stress is defined as the imbalance between cellular pro-oxidant levels and reducing capacity due to excessive reducing capacity (Xiao and Loscalzo, 2020[290]). Reductive stress is characterized by depletion of basal ROS levels due to an increase in NAD(P)H/NAD^+^ and GSH/GSSG redox couples, the main cellular redox buffers, and increased cellular maximal ReBC and/or overexpression of antioxidant enzymatic systems (Pérez-Torres et al., 2017[209]; Xiao and Loscalzo, 2020[290]; Zhang and Tew, 2021[308]).

The studies on reductive stress have reported that increases in NADPH/NADP^+^ or/and GSH/GSSG production or decreases in their consumption cause reductive stress. In these studies, increases in their production of them were associated with reasons such as glucose-6-phosphate dehydrogenase (G6PD) overexpression, Nrf2 activation, heat shock protein 27 (Hsp27) overexpression, γ-glutamylcysteine ligase (GCL) overexpression and lamin C mutations (Pérez-Torres et al., 2017[209]; Xiao and Loscalzo, 2020[290]; Xiao et al., 2018[292]). Decreases in consumption of them are associated with reasons such as overexpression of the dominant negative mutant of NOX4 (DN-NOX4; loss of NOX4 activity) (Pérez-Torres et al., 2017[209]; Xiao and Loscalzo, 2020[291]). In addition, stressful situations such as exogenous addition of mitochondrial complex I substrates, hypoxia, nicotinamide nucleotide transhydrogenase (NNT) reversal, NNT inactivation and reverse electron transfer (RET) lead to cause reductive stress increase in mitochondrial NADH/NAD^+^ (Pérez-Torres et al., 2017[209]; Xiao and Loscalzo, 2020[291]; Xiao et al., 2018[292]).

Paradoxically, reductive stress is also associated with oxidative stress. Chronic reductive stress can induce oxidative stress through stimulating ROS production (Gores et al., 1989[91]; Korge et al., 2015[140]; Singh et al., 2015[247]; Yu et al., 2014[300]). For example, it has been reported that chronic reductive stress created by long-term stimulation with N-acetyl-L-cysteine (NAC) stimulates mitohormesis, an adaptive response that regulates mitochondrial functions by stimulating ROS production. In addition, it was stated that the dose and duration of antioxidant application in reductive stress may have an effect on the response in the cell (Singh et al., 2015[247]).

Overexpression of Nrf2 is known to induce reductive stress, but the effect of Nrf2 activation in cardiac pathologies is controversial. While there is evidence that Nrf2 can ameliorate cardiac pathology (Ashrafian et al., 2012[4]; Cao et al., 2015[30]; Strom and Chen, 2017[254]; Zhu et al., 2008[316]), it has also been associated with the progression of various cardiac pathologies (Bhide et al., 2018[19]; Guan et al., 2019[94]; Kannan et al., 2013[126]; Rajasekaran et al., 2011[223]). Therefore, it is clear that future studies are needed to investigate how reductive stress affects cell metabolism and how cells adapt their metabolism to reductive stress (Audousset et al., 2021[5]; Ma et al., 2020[172]; Pérez-Torres et al., 2017[209]; Xiao and Loscalzo, 2020[290]). Stress responses, such as Keap1-dependent and Keap1-independent regulation of Nrf2, are often controlled by ubiquitylation, a modification whose specificity is conferred by E3 ligases (Manford et al., 2020[176]). A recent study showed that Cullin 2 fem-1 homolog B (CUL2^FEM1B^) , a ubiquitin E3 ligase, targets reduced Folliculin-interacting protein 1 (FNIP1), which can alleviate reductive stress caused by excessive antioxidant processes and promote physiological ROS (Manford et al., 2020[176], 2021[175]).

It is clear that further research is needed to fully elucidate the reductive stress mechanism and cellular reductive stress response. However, different studies to date reveal the devastating effects of reductive stress: 1) Disrupt ROS-related signaling pathways. 2) Alter disulfide bond formation in proteins, thereby causing activation of the unfolded protein response and ER stress. 3) Reduce metabolism 4) Disrupt mitochondrial homeostasis (Pérez-Torres et al., 2017[209]; Xiao and Loscalzo, 2020[290]; Zhang and Tew, 2021[308]). Although studies have generally focused on the relationship between mitochondrial dysfunction and oxidative stress, it has been shown that reductive stress may also cause mitochondrial dysfunction (Ma et al., 2020[172]; Peris et al., 2019[210]; Singh et al., 2015[247]). For example, reductive stress has been shown to trigger mitochondrial dysfunction and cytotoxicity in cultured cells (Zhang et al., 2012[307]). Similar to oxidative stress, reductive stress is associated with numerous disease pathophysiology due to impaired redox homeostasis (reviewed in detail and reported by (Bellezza et al., 2020[16]; Manford et al., 2021[175]; Pérez-Torres et al., 2017[209]; Vardar Acar et al., 2021[278]; Xiao and Loscalzo, 2020[290]; Zhang and Tew, 2021[308])) (Figure 6(Fig. 6)).

### Dose is everything: the poison or the medicine

ROS were initially thought to be potentially harmful by-products of aerobic metabolism and were often associated with the principle of oxidative stress, that induces pathology by causing damage to biomolecules. However, with the emergence of non-toxic levels of ROS also serving as signaling molecules to regulate biological and physiological processes, and understanding the details of redox homeostasis and stress, it became clear that the concepts of ROS and stress should be approached from a broad perspective (Görlach et al., 2015[92]; Lu et al., 2021[165]; Schieber and Chandel, 2014[232]; Sies et al., 2017[243]). Accumulating evidence indicates that most stressors, including ROS, exert a biphasic dose-dependent effect on health. In other words, high levels and long-term exposure to stress factors such as ROS may be harmful to cell and organism health, while low-level exposure will be beneficial (Lu et al., 2021[165]; Lushchak and Storey, 2021[170]; Zhou et al., 2019[314]).

**Figure 7:**
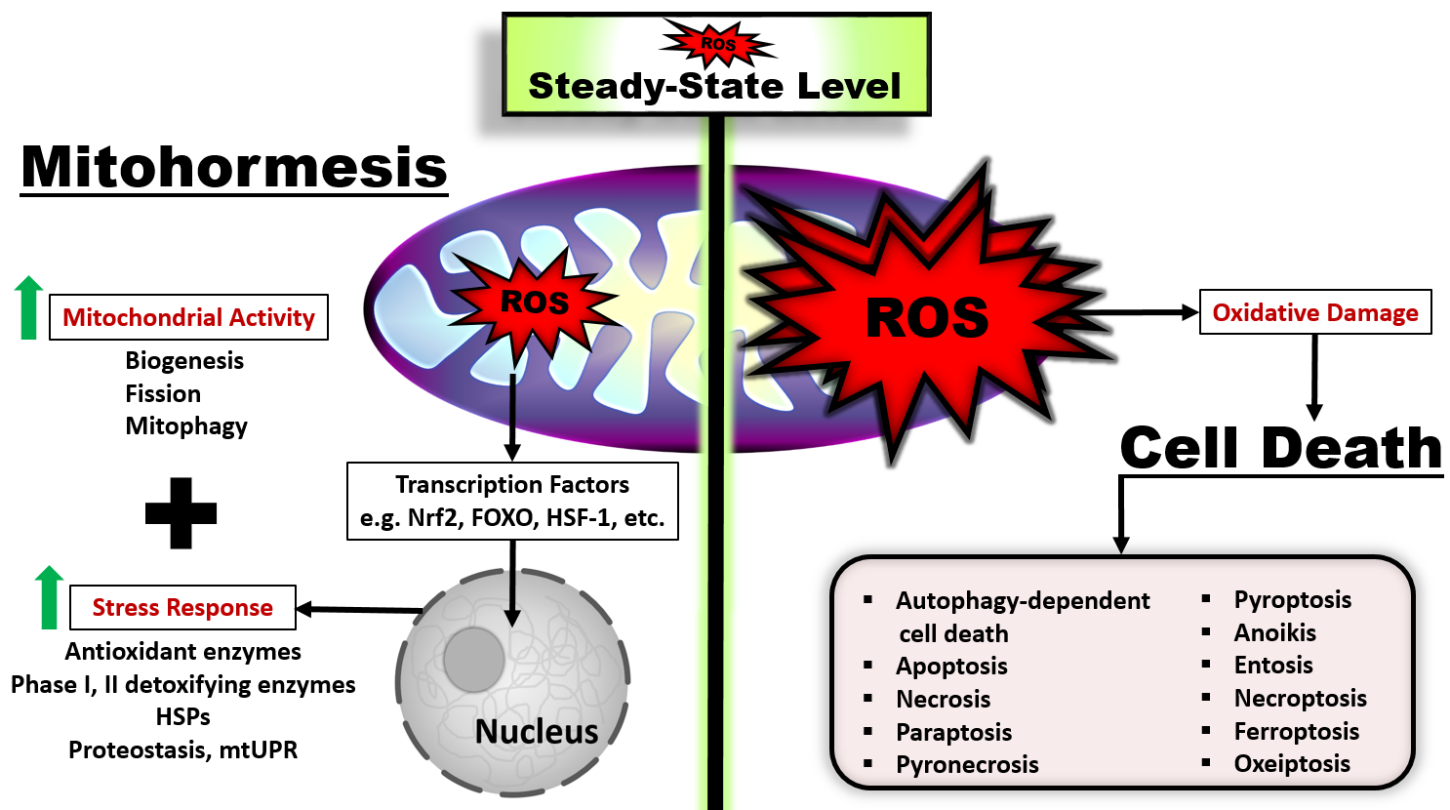
The levels of ROS determine the decision of the cells between survival and death. Mitohormesis is a pro-survival adaptive response that results in increased health and vitality in a cell, tissue, or organism through mild ROS levels released by the induction of reduced amounts of mitochondrial stress. To maintain cell survival, the mild levels of ROS induce the activation of a retrograde mitochondrial-nucleus signaling mechanism, leading to a stress response in the cell and causing an increase in mitochondrial activity. High levels of ROS act as second messengers that cause different cell death mechanisms to be engaged in the cells.

High levels of ROS accompany a wide variety of disease pathophysiologies, causing extensive and irreparable cellular damage and cell death (Ghosh et al., 2017[85]; Sharifi-Rad et al., 2020[235]). Increasing evidence suggests that ROS also play a critical role as signaling molecules for cell death pathways (Ghosh et al., 2017[85]; He et al., 2017[105]; Jia et al., 2021[119]; Villalpando-Rodriguez and Gibson, 2021[280]) (Figure 7(Fig. 7)). In general, the roles of ROS in necrosis, apoptosis and autophagy death pathways, known as the most common forms of cell death, have been emphasized, but many new cell death methods have been defined in recent years (Ghosh et al., 2017[85]; He et al., 2017[105]; Jia et al., 2021[119]; Nirmala and Lopus, 2020[196]; Tang et al., 2019[262]; Villalpando-Rodriguez and Gibson, 2021[280]; Yan et al., 2020[295]). For example, paraptosis, characterized by extensive cytoplasmic vacuolization derived from the enlarged ER or mitochondria, has been shown in a number of studies to be associated with ROS production, accumulation of misfolded proteins in the ER, and mitochondrial Ca^2+^ overload (Gandin et al., 2012[80]; Ghosh et al., 2016[84]; Lee et al., 2016[152]; Shiau et al., 2017[238]; Sperandio et al., 2000[250]; Yoon et al., 2014[299]). Autophagy-dependent cell death can be presented as an example of another death pathway induced by ROS. Studies continue on autophagy-dependent cell death mechanisms, a regulated form of cell death that is mechanically dependent on the autophagic machinery (or its components) (Doherty and Baehrecke, 2018[66]; Galluzzi et al., 2018[76]). In a study with mitochondrial complex I and II inhibitors, it was shown that autophagic cell death mediated by ROS production was induced in HEK 293, U87 and HeLa cells (Chen et al., 2007[37]). Beside the existence of different types of cell death is now known, the general consensus is that ROS signaling and control is the dominant common feature between different types of cell death inducing specific cell signaling pathways (Villalpando-Rodriguez and Gibson, 2021[280]).

It is the most simplified expression of the relationship between cell death pathways and ROS levels: very high levels of ROS cause necrosis, high levels of ROS cause apoptosis, and non-lethal doses of ROS cause autophagy (Ghosh et al., 2017[85]; Jung et al., 2020[124]; Zhou et al., 2019[314]). Although this statement is partially true, it is clear that there is a much more complex relationship between ROS levels and cell death pathways. In light of current research, important information has been obtained about the relationship between ROS and cell death pathways: 1) Oxidative damage is both a reason and a consequence of various cell deaths. 2) Not only the level of ROS produced but also the type of ROS can determine the ability of cells to undergo cell death. 3) The increase in ROS levels is both a consequence of cell death and a key player in inducing cell death. 4) The different types of cell death are not completely independent processes. There is a crosstalk between different types of cell death. ROS may also play a role in the crosstalk between different types of cell death. 5) Cell organelles play a crucial role in organizing ROS induction of different types of cell death (Villalpando-Rodriguez and Gibson, 2021[280]). It is clear that further studies are needed to elucidate this comprehensive and complicated relationship between elevated ROS levels and cell death pathways.

In contrast to high levels of ROS, low/mild levels of ROS are one of the most important factors that act as signaling molecules for cells to activate their survival pathways by avoiding being directed to death pathways. These mild levels of ROS (non-cytotoxic concentration) produced in mitochondria initiate a series of cellular events that protect cells from harmful effects and promote health and vitality, a process known as "mitohormesis" (Ristow and Zarse, 2010[229]; Tapia, 2006[264]) (Figure 7(Fig. 7)). This process is performed very sensitively and by providing the regulation of mitochondrial functions, a signal is created for the continuation of cell homeostasis with mitohormesis in the cell: biogenesis (to increase the number of mitochondria and active respiratory components), mitophagy (to dispose of damaged units) and fission (to enhance mitochondrial membrane potential (ΔΨ) and ATP production-the number of mitochondria is increased, while the surface area per unit is decreased) (Palmeira et al., 2019[205]). In addition to enhancing mitochondrial biogenesis (Cox et al., 2018[48]; Hamilton and Miller, 2017[99]) and improving mitochondrial function (Hamilton and Miller, 2017[99]; Miller et al., 2012[186]; Schulz et al., 2007[234]; Wolff et al., 2020[287]), mitohormesis may also contribute to health benefits (Bárcena et al., 2018[10]; Ristow and Schmeisser, 2014[228]) and promote life span extension (Bárcena et al., 2018[10]; Ristow and Schmeisser, 2014[228]) by upregulating antioxidant enzymes (Cox et al., 2018[48]), improving redox homeostasis (Cox et al., 2018[48]), promoting protein folding (Gariani et al., 2016[81]; Ristow and Schmeisser, 2014[228]) and protecting proteome integrity (Ristow and Schmeisser, 2014[228]).

Mild perturbations in mitochondrial function due to various cellular stress factors such as calorie restriction, hypoxia, physical activity, glucose restriction, decreased insulin/IGF-1 signaling, and ROS-inducing compounds can mediate an increase in mitochondria-derived ROS and stimulate mitohormesis (Fischer and Ristow, 2020[72]; Ristow and Schmeisser, 2014[228]). These mild ROS levels induce activation of a retrograde mitochondria-nucleus signaling mechanism (Fischer and Ristow, 2020[72]). Thus, transcription of several genes involved in the cellular stress response, such as antioxidant enzymes, stress proteins, and mitochondrial unfolded protein response (UPRmt), is stimulated by redox-sensitive transcription factors such as Nrf2, FOXO, and heat shock factor 1 (HSF-1) (Bárcena et al., 2018[10]; Ristow and Schmeisser, 2014[228]) (Figure 7(Fig. 7)).

Much of the information we have learned about mitohormesis is based on model organisms, especially *C. elegans* (Miller et al., 2018[187]; Ristow and Schmeisser, 2014[228]; Tian et al., 2023[267]; Yun and Finkel, 2014[301]). Although this process has not yet been extensively studied in higher-level organisms, current studies promise hope to elucidate the roles of mitohormesis in maintaining human health (Chirumbolo et al., 2022[39]; Gohel and Singh, 2021[89]; Kostyuk et al., 2022[142]; Singh et al., 2015[247]; Suárez-Rivero et al., 2022[255]; Vardar Acar et al., 2021[278]). For example, accumulating evidence indicates that mitohormesis can restore cellular homeostasis by activating mitochondrial biogenesis, redox homeostasis, mitochondrial function, and antioxidant enzymes under chronic reductive stress (Bárcena et al., 2018[10]; Cox et al., 2018[48]; Fischer and Ristow, 2020[72]; Palmeira et al., 2019[205]; Ristow and Schmeisser, 2014[228]; Singh et al., 2015[247]; Spanidis et al., 2018[249]; Yun and Finkel, 2014[301]). In the study including propionic acidemia, mitochondrial diseases and mucopolysaccharidosis IV diseases from IEMs, data compatible with reductive stress were obtained in these diseases, but mitohormesis was noted with near-normal mitochondrial membrane potential and high intracellular ATP measurement results (Vardar Acar et al., 2021[278]). Therefore, it should not be forgotten that mitohormesis may be a pro-survival response accompanying the disease pathophysiology in diseases with chronic cellular stress. In another example, it has been reported that tetracycline, an antibiotic, activates the mitochondrial homeostasis balancing pathway, UPRmt, thereby reducing the pathogenicity of the disease-associated mutation and improving mitochondrial function. As a result, it has been suggested that there may be a new therapeutic approach based on mitohormesis beyond the traditional treatment used against mitochondrial diseases (Suárez-Rivero et al., 2022[255]). These studies indicate that a comprehensive elucidation of the mitohormesis process may lead to both a better understanding of the pathophysiology of diseases and the development of effective treatments in which an adaptive response can be created by activating the mitohormesis process.

## Conclusion

Cellular redox homeostasis, defined as the balance between pro-oxidants, especially ROS, and antioxidant capacity, is a crucial importance for the maintenance of vitality. ROS, kept at steady-state level in the cell under physiological conditions, is an important signaling molecule and provides the structural and functional integrity of the cell by taking part in many cellular pathways that are beneficial for the organism. Redox balance is tightly controlled by enzymatic and non-enzymatic antioxidant systems, and directly or indirectly via many enzymes and transcription factors. However, the direct or indirect disruption of redox homeostasis causes oxidative and reductive stress, called redox stress, resulting in the deterioration of the redox signal and its control. Oxidative and reductive stress are associated with many disease pathophysiologies. While oxidative stress, characterized by high ROS levels, causes extensive and irreparable cellular damage and cell death, reductive stress, characterized by low ROS levels, is harmful at least as oxidative stress, because of impairing physiological cell functions. However, non-toxic levels of ROS (mild levels of ROS) serve as signaling molecules to regulate biological and physiological processes and are involved in the regulation of redox homeostasis. Ensuring the regulation of cellular redox homeostasis affects the course of health and disease states by affecting from tissue to organism integrity. For this reason, the follow-up of the existing cellular redox status before, during and after the treatment, regardless of the type of disease, might be a guide in monitoring of the response to the treatment and the course of the disease. In addition, ensuring the regulation of ROS intensity in an appropriate, correct and controlled manner has the potential to be a parameter that can be used directly as a therapeutic, beyond the preventive treatment effect in the treatment processes of diseases: Ingenuity is hearing the sound of the cells.

## Declaration

### Declaration of competing interest

The authors declare that they have no conflict of interest.

### Author contributions

N.V.A and R.K.Ö. devised the main conceptual ideas. N.V.A. wrote the initial draft of the manuscript and prepared the table and figures. R.K.Ö. contributed to writing the initial draft of the manuscript, edited and reviewed the manuscript. All of the authors have read and approved the final version submitted.

## Figures and Tables

**Table 1:**
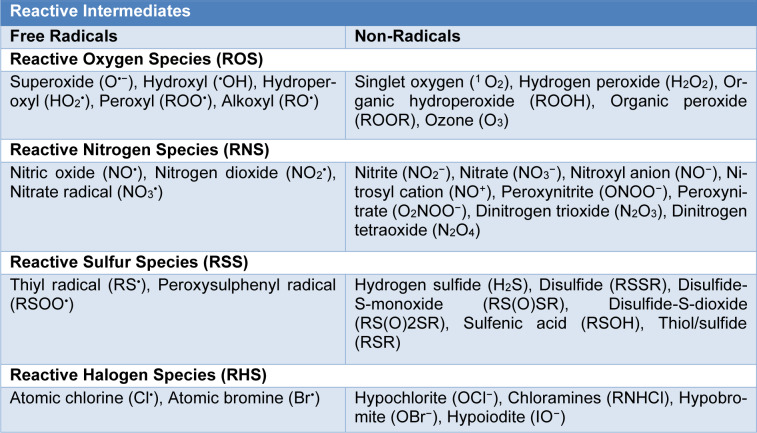
Instance of free radical and non-radical species [Phaniendra et al., 2015; Martemucci et al., 2022; Tanaka and Vécsei, 2020]

**Figure 1:**
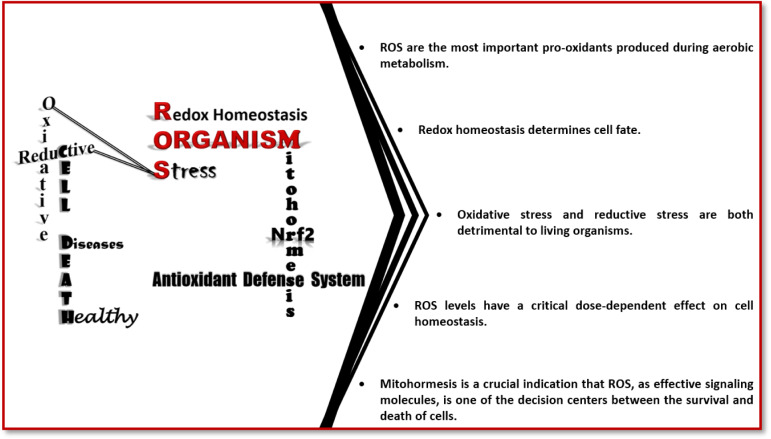
Graphical abstract
